# Tau missorting and spastin-induced microtubule disruption in neurodegeneration: Alzheimer Disease and Hereditary Spastic Paraplegia

**DOI:** 10.1186/s13024-015-0064-1

**Published:** 2015-12-21

**Authors:** Hans Zempel, Eva-Maria Mandelkow

**Affiliations:** German Center for Neurodegenerative Diseases (DZNE), Bonn, Germany; CAESAR Research Center, Bonn, Germany; MPI for Metabolism Research, Hamburg Outstation, c/o DESY, Hamburg, Germany

**Keywords:** Tau, Spastin, TTLL6, Microtubule, Amyloid-beta, Alzheimer disease, Hereditary spastic paraplegia, Neurodegeneration, Cell polarity

## Abstract

In Alzheimer Disease (AD), the mechanistic connection of the two major pathological hallmarks, namely deposition of Amyloid-beta (Aβ) in the form of extracellular plaques, and the pathological changes of the intracellular protein Tau (such as phosphorylation, missorting, aggregation), is not well understood. Genetic evidence from AD and Down Syndrome (Trisomy 21), and animal models thereof, suggests that aberrant production of Aβ is upstream of Tau aggregation, but also points to Tau as a critical effector in the pathological process. Yet, the cascade of events leading from increased levels of Aβ to Tau-dependent toxicity remains a matter of debate.

Using primary neurons exposed to oligomeric forms of Aβ, we have found that Tau becomes mislocalized (missorted) into the somatodendritic compartment. Missorting of Tau correlates with loss of microtubules and downstream consequences such as loss of mature spines, loss of synaptic activity, and mislocalization of mitochondria.

In this cascade, missorting of Tau induces mislocalization of TTLL6 (Tubulin-Tyrosine-Ligase-Like 6) into the dendrites. TTLL6 induces polyglutamylation of microtubules, which acts as a trigger for spastin mediated severing of dendritic microtubules. Loss of microtubules makes cells unable to maintain transport of mitochondria, which in turn results in synaptic dysfunction and loss of mature spines. These pathological changes are absent in TauKO derived primary neurons. Thus, Tau mediated mislocalization of TTLL6 and spastin activation reveals a pathological gain of function for Tau and spastin in this cellular model system of AD.

In contrast, in hereditary spastic paraplegia (HSP) caused by mutations of the gene encoding spastin (spg4 alias SPAST), spastin function in terms of microtubule severing is decreased at least for the gene product of the mutated allele, resulting in overstable microtubules in disease model systems. Whether total spastin severing activity or microtubule stability in human disease is also affected is not yet clear. No human disease has been associated so far with the long-chain polyglutamylation enzyme TTLL6, or the other TTLLs (1,5,11) possibly involved.

Here we review the findings supporting a role for Tau, spastin and TTLL6 in AD and other tauopathies, HSP and neurodegeneration, and summarize possible therapeutic approaches for AD and HSP.

## Background

In Alzheimer Disease (AD), one observes two major aggregating proteins: Amyloid-beta (Aβ), which accumulates in the form of plaques mainly in the extracellular space and the microtubule associated protein Tau, which aggregates in the form of paired helical filaments (PHFs) intracellularly [59]. The connection of these two major pathological hallmarks is not well understood. Evidence from AD, Down Syndrome (Trisomy 21), and from corresponding animal models, indicates that aberrant production of Aβ is upstream of Tau pathology. In genetic cases of AD, Aβ deposition precedes Tau aggregation. Additionally, there is no Tau mutation that causes AD or even increases the risk of getting AD. Genetic cases of AD cause a net increase in Aβ production or a shift in the isoform ratio towards more aggregation prone forms of Aβ, or increase the aggregation propensity of Aβ itself, as in the case of the arctic mutation [2, 23, 39, 49]. Conversely, a mutation that decreases the aggregation capacity of Aβ is protective of AD [33].

Nonetheless, Tau aggregates, but not Aβ-plaque burden, correlate with synaptic loss and cognitive decline in AD patients [7]. Also, Tau-knockout (TauKO) protects from AD-like pathologies in several models of AD [30, 54, 78]. Despite the fact that there is no genetic association of Tau with AD, mutations of Tau cause different forms of neurodegenerative disease (e.g. Frontotemporal Dementia with Parkinsonism linked to Chromosome 17 (FTDP-17) [29, 51]), or increase the risk for FTD-spectrum disorders [40]. Tau pathology is also an important part of many other neurodegenerative diseases (FTLD-Tau, Parkinsonism, ALS, PSP, Tangle-only dementia, CTE etc.; for review see [15, 65]). These observations point to Tau as a critical effector in pathological processes.

Like Tau, spastin is an important regulator for microtubule dynamics. In contrast to Tau, which stabilizes microtubules, spastin’s main function is to sever microtubules. Thereby spastin either reduces microtubule mass, generating short microtubules for the seeding of new microtubules, or facilitates translocation of microtubules. Mutations of spastin causing Hereditary Spastic Paraplegia (HSP) often compromise the severing ability of spastin, which is considered a loss of function effect. However, microtubule binding of spastin often is unaffected by these mutations. Spastin therefore might bind and bundle microtubules, which can be considered a pathological gain of function (for review on SPAST caused HSP see [61]).

We recently discovered a pathological cascade that involves missorting of the axonal microtubule associated protein Tau (MAPT) into the somatodendritic compartment of neurons. This causes mislocalization of TTLL6 into dendrites, polyglutamylation of microtubules, and finally microtubule severing of dendritic microtubules by spastin. The implication of this pathological cascade for AD, FTLD, HSP, and other forms of neurodegeneration is not clear. Here we summarize possible ties and differences of Tau and spastin action in neurodegeneration, and outline possible therapeutic approaches.

## Review

### The pathological cascade: from Amyloid-β exposure via Tau missorting to spastin activation

To elucidate the pathological cascade leading to neurodegeneration in AD, we established a system to model the effects of Aβ on Tau. In mature rodent primary neurons, Tau is present in several isoforms, is sorted into the axon and excluded from the somatodendritic compartment, all of which is similar to the adult human brain. To trigger AD-like pathological changes of Tau, we exposed primary neurons to a pre-aggregation form of Aβ (oligomeric Aβ), which is thought to be the toxic trigger in AD [3, 24, 35]. We investigated changes of Tau and related cellular pathologies using biochemical methods, fluorescence microscopy including live cell imaging, and electron microscopy.

After exposure to oligomeric Aβ, Tau becomes mislocalized (missorted) into the somatodendritic compartment, a feature reminiscent of incipient AD [5]. Interestingly, the missorted Tau does not originate from the axon, but is newly synthesized, fails to become sorted into the axon, and instead spreads throughout the somatodendritic compartment. Missorting of Tau correlates with a loss of synapses, most pronounced in dendrites containing high amounts of Tau [81]. This provides a link between the mislocalization of Tau and the cognitive decline observed in mouse models of AD and in AD patients. One of the key features is a dramatic loss of microtubules in neurons with missorted Tau [80]. Microtubules are of great importance as tracks of the intracellular traffic system based on microtubule-dependent motor proteins. This is particularly so for neurons whose extended processes require highly efficient transport.

**Fig. 1:**
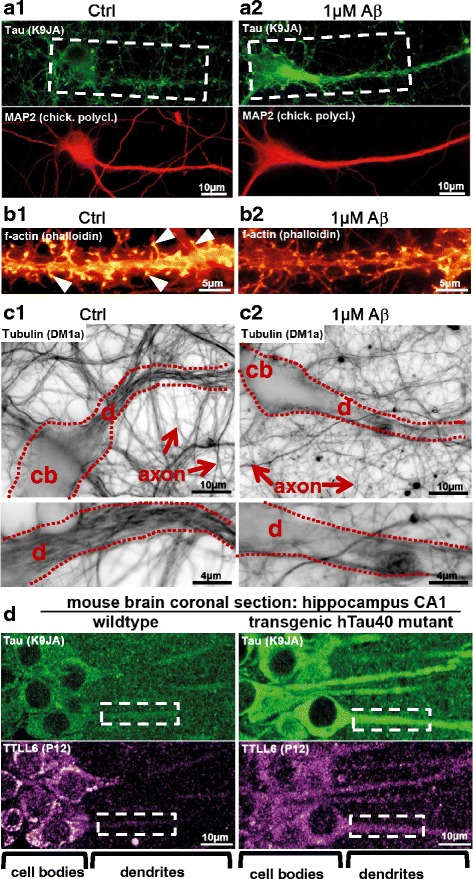
Aβ oligomers induce missorting of Tau into dendrites. Missorting of Tau correlates with dendritic loss of microtubules and mislocalization of TTLL6. **a**, **b** Micrographs of fluorescently stained primary hippocampal neurons aged for 21 days in vitro after treatment with Aβ oligomers. (**a**) Tau is stained with an antibody against total Tau (K9JA, green color). Staining of the dendritic marker MAP2 is shown in red. In control conditions, Tau and MAP2 do not colocalize (a1, boxed area shows a dendrite and cell body without Tau). After exposure to Aβ, Tau becomes missorted into the somatodendritic compartment, where it then colocalizes with MAP2 (a2, boxed area shows a dendrite and cell body with Tau missorting). (**b**) Staining of f-actin in dendritic segments with phalloidin. In control conditions, there are numerous spines (b1, f-actin positive dendritic protrusions, arrowheads). After exposure to Aβ, mature spines are lost (b2). **c** Microtubules were stained using an antibody against tubulin after fixing and extracting to favor microtubules over free tubulin. The filamentous structures within the dendrite in c1 (framed by dotted lines) are microtubules. In control conditions dendrites display dense microtubules (c1), whereas microtubules are lost after Aβ treatment (c2). Proximal parts of dendritic segments are magnified in lower panels. cb: cell body; d: dendrites. **d** Brain sections from Tau transgenic mice where missorting of Tau is present, TTLL6 is mislocalized to dendrites even without exposure to Aβ. Boxed areas highlight examples of dendritic segments. Adapted from [78]

Treatment of neurons with Aβ oligomers leads to missorting of Tau into dendrites, with subsequent decay of microtubules and mature spines (Fig. 1a, b, c). Consequently, mitochondria, vesicles, and other cargo suffer from traffic jams in these dendrites. Neurofilaments that are usually transported along microtubules from the soma into the axon, become also mislocalized to the somatodendritic compartment, which indicates impaired transport.

Most of these pathological changes depend on the presence of Tau, as in neurons derived from Tau knockout mice (TauKO), loss of microtubules and synapses, aberrant mitochondria and neurofilament distribution do not occur [78].

In search for the causes of microtubule breakdown in dendrites, we found that the decay of microtubules is executed by spastin, a microtubule severing enzyme. Spastin in turn is recruited to microtubules by polyglutamylation of microtubules, conferred by the enzyme TTLL6 (Tubulin-Tyrosin-Ligase-Like-6) and possibly related isoforms. TTLL6 is a microtubule modifying ligase which catalyzes the addition of polyGlu residues to Glu residues near the C-terminal tail of α-tubulin. This highly acidic configuration leads to the recruitment of the microtubule severing protein spastin and subsequent destruction of microtubules. TTLL6 is mislocalized from the soma into dendrites by a Tau dependent mechanism, likely involving an interaction of TTLL6 with the N-terminal half of Tau [78] (Fig. 1d) [38].

**Fig. 2:**
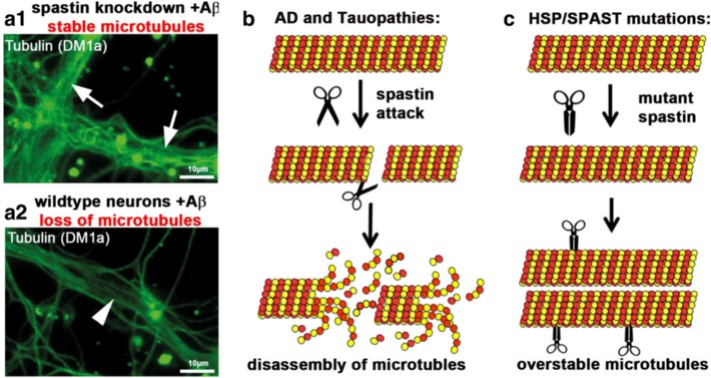
Spastin executes microtubule loss after invasion of dendrites by Tau and TTLL6. **a** Experimental evidence for spastin mediated microtubule breakdown in a neuronal cell model of AD. Spastin was silenced using shRNA with a vector co-expressing mRFP in primary hippocampal neurons aged 16 days in vitro. Cells were then treated with 1 μM Aβ for 3 h. Silencing of spastin results in stable microtubules after Aβ treatment. Cells expressing shRNA show no microtubule reduction in dendrites (arrows, a1). Neighboring untransfected cells with normal spastin levels show loss of microtubules in dendrites after Aβ treatment (a2, arrowheads). **b**, **c** Diagrams of proposed mechanisms for microtubule breakdown in AD and overstable microtubules in HSP. (**b**) In Alzheimer disease and other tauopathies, spastin mediates microtubule disassembly in dendrites in the presence of missorted Tau. (**c**) In the case of HSP caused by mutations of *sp4/SPAST*, spastin is unable to sever microtubules, but might still be able to bind them, resulting in overstable and bundled microtubules. Modified from [32, 78]

Normal spastin levels seem to be essential for the microtubule breakdown induced by Tau missorting and Aβ-exposure. In control neurons, Aβ exposure leads to microtubule destruction, while knockdown of spastin via shRNA results in stable microtubules in spite of Aβ exposure (Fig. 2a). Of note, once the toxic stimulus of Aβ-oligomers disappears, microtubule density recovers, implying that without constant or repetitive exposure to toxic, oligomeric forms of Aβ, spastin over-activity returns to baseline levels without further intervention [78]. Thus, spastin is the effector of microtubule breakdown in this cell culture model of Alzheimer disease (Fig. 2b). The flip side of the spastin coin is the suggested mechanisms of HSP, where mutant or absent spastin activity impairs normal microtubule dynamics (see below), which results in bundled and overstable microtubules (Fig. 2c) [32].

Apart from the scenario described above, Tau has been implicated in several ways in the impairment of microtubules and synapses. Along with the related proteins MAP2 and MAP4, Tau belongs to the class of "structural MAPs" capable of binding to and stabilizing microtubules. Proper axonal sorting of Tau depends on intact Tau-microtubule interactions [34]. In the pathological condition of Tau missorting, however, Tau is altered by posttranslational modifications, notably phosphorylation at a number of sites [25, 48]. In contrast to low-level physiological phosphorylation, pathologically increased phosphorylation could lead to detachment of Tau from microtubules, with subsequent destabilization of microtubules (for discussion of physiological vs. pathological phosphorylation see [1, 77, 79]). An example is the phosphorylation of Tau at the KXGS-motifs in the repeat domain of Tau [71, 81]. These sites regulate the binding of Tau to microtubules and are targets of the Microtubule-Affinity-Regulating Kinase (MARK). In maturing neurons MARK is most active in growth cones, in the soma and the dendrites, but not in axons [72]. When phosphorylated at the KXGS-motifs, Tau cannot bind and stabilize microtubules, and can become missorted [43]. This in turn can lead to abberant interactions of missorted Tau with proteins in the somatodendritic compartment, e.g. the tyrosine kinase Fyn [30].

Tau has been shown to protect microtubules against attack by another microtubule severing protein, katanin [52]. Thus, detachment of Tau from microtubules as a result of Tau phosphorylation could further promote the destabilization of microtubules, either by lack of stabilization and/or by loss of protection against severing. Tau would then also be free to translocalize into spines and mediate the loss of synapses, as shown by experiments using pseudophosphorylated or mutated Tau [28, 78]. The precise mechanism of how phosphorylated Tau induces loss of mature spines and loss of f-actin in spines is unclear. Suggested mechanisms circle around a NMDA-receptor mediated calcium rise, as glutamate triggers similar pathological effects [81]. For example, NMDA-receptors could be released from the synapse to the dendritic shaft after f-actin disassembly mediated by phosphorylated Tau. This would cause long term depression and excitotoxicity, possibly by a calcineurin dependend mechanism [30, 57, 74, 79].

### Spastin activation in AD vs. spastin inactivity in HSP

**Fig. 3:**
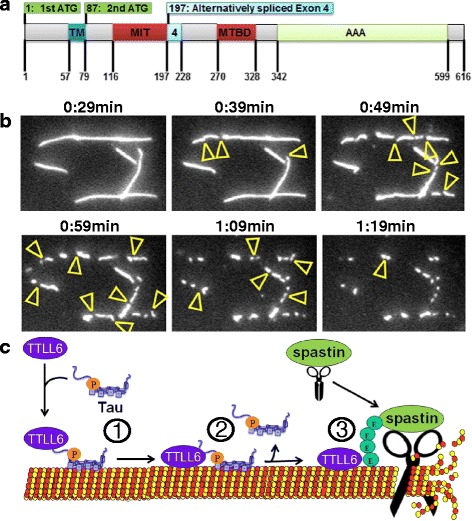
Spastin domain structure, and proposed mechanism of Tau mediated microtubule loss. **a** Domain structure of spastin. Spastin contains four main domains: TM, transmembrane domain; MIT, microtubule interacting and transport domain, MTBD, microtubule binding domain; and AAA, ATPase associated with various cellular activities domain. Adapted from [56, 60]. **b** Severing of microtubules by spastin and subsequent disassembly. MTs are fluorescently labeled (Atto488) and stabilized by taxol. Yellow arrowheads indicate areas where microtubules are severed by spastin. Figure by courtesy of G. Woehlke (Tech. Univ. Munich), adapted from [17]. For movies of spastin-induced severing of microtubules see [17]. **c** Model of Tau dependent spastin mediated severing of microtubules: (1) Missorted Tau induces translocation of TTLL6 to dendritic microtubules. (2) Tau detaches from microtubules (i.e. has a reduced dwell time on microtubules, possibly as a result of increased phosphorylation by MARK). (3) TTLL6 executes polyglutamylation of microtubules which triggers the activation of spastin and severing of microtubules

Mutations in the gene encoding spastin –SPAST– are the single most common cause of autosomal dominant hereditary spastic paraplegia (HSP) and cause about 40 % of familial HSP and about 20 % of sporadic HSP (for recent review on HSP see [21]). Spastin protein occurs in four isoforms (Fig. 3a), depending on alternative splicing of exon 4 and an alternative translation initiation codon, but the predominant isoforms are of 60 and 68 kDa size [11].

Spastin has diverse cellular functions. They include membrane shaping and membrane trafficking, but the best known function is microtubule severing. Spastin consists of two main structural domains (Fig. 3a): (i) a microtubule interacting and transport (MIT) domain at the N-terminus, and (ii) an AAA-ATPase domain at the C-terminus, which is responsible for microtubule severing (Fig. 3b). Attached to the AAA-ATPase domain is the microtubule binding domain (MTBD), thus spastin has two domains interacting with microtubules. Near the N-terminus there is also a transmembrane (TM) domain possibly involved in ER shaping and trafficking. Only the full length isoform (M1) with 616aa contains the TM domain, while the slightly shorter isoform M87 (M85 in rodents, hereafter referred to as M87), lacks the highly hydrophobic 86 N-terminal aa, which include the TM domain [11].

In HSP with SPAST mutations the MT-severing activity of spastin is decreased: Over 300 pathological mutations, including partial deletions, have been described, mostly in the AAA-ATPase domain [60]. In SPAST related HSP, haploinsufficiency of spastin results in impaired microtubule severing and as such represents a loss of function phenotype. As the MIT or the MTBD domain in HSP-patients is still intact, spastin mutants can still bind to and crosslink microtubules, which could result in bundles of stable microtubules [62]. This represents a gain of pathologic function (see below).

Thus, in a formal sense, the view that microtubule severing by spastin might be impaired in HSP (i.e. under-activity of spastin) is the opposite to our proposed pathological cascade of AD, which involves aberrant microtubule breakdown by spastin (i.e. over-activity of spastin; Fig. 2b, c, Fig. 3c). Under- and over-activity of spastin are the two sides of the same coin, both leading to neurodegeneration. In the following sections, we will explore common mechanisms and potential therapeutic avenues.

### Triggering spastin activity: roles for TTLLs

Spastin is recruited to microtubules by polyglutamylation, a specific type of posttranslational modification of microtubules [38]. Accordingly, we found that the polyglutamylation of microtubules precedes Aβ-induced microtubule loss. Tau appears to mediate the activation of spastin (=microtubule severing) via recruitment of TTLL6. Tau is necessary for an enrichement of TTLL6 into dendrites, where TTLL6 then catalyzes the ligation of polyGlu residues within the C-terminal tail of α-tubulin.

Polyglutamylation can also be induced by other members of the Tubulin-Tyrosin-Ligase-Like family, in particular TTLL1, 4, 5, 6, 7, 11, and 13 [31]]. Of these, mainly TTLL1, 4, 5 and 7 initiate polyglutamylation with different preferences for α-tubulin and β-tubulin. The main glutamylation sites are on Glu445 of α-tubulin (type A1A/B), and on Glu435 of β-tubulin (type B2A/B, Glu438 for B3-tubulin), but several other sites are possible due to the high glutamate content of the acidic C-terminal tail of tubulin [31]. TTLL4 or 7 catalyze the ligation of further Glu residues to microtubules, preferentially on β-tubulin, and TTLL5 on α-tubulin. This results in branching points and short polyGlu side chains that do not yet suffice for spastin mediated severing of microtubules. By contrast, only TTLL6 and 11 elongate polyGlu side chains on α-tubulin, in a manner which initiates spastin mediated severing [38].

It is currently not clear whether Tau-dependent mislocalization of TTLL6 in response to Aβ is the sole mediator of microtubule loss in dendrites, or whether this can be achieved by other TTLLs as well. Polyglutamylation of microtubules is reversible; the enzymes that are responsible for deglutamylation have recently been identified and belong to the class of cytosolic carboxypeptidases (CCP). Of these, CCP5 removes mainly branching points, while CCP1, 4, 6 reduce the glutamate chain length [55]. Thus, other polyglutamylases and deglutamylases could also be involved in Aβ and Tau mediated loss of microtubules.

### Tackling pathological spastin activity: therapeutic potential for AD and HSP

The proposed mechanisms for the pathological changes in spastin activity in HSP and in our neuronal cell model of AD are flip sides of the same coin. In HSP and models thereof, spastin activity is decreased, whereas in the cell model of AD, spastin is aberrantly activated and induces breakdown of microtubules. Consistent with this, AD patients and several model systems of tauopathy show decreased microtubule density (see Table 1). Thus, inhibiting the microtubule severing activity of spastin could be beneficial in an AD setting. In HSP caused by SPAST gene mutations, spastin is unable to sever microtubules, but at least some forms of mutated spastin might still bind and cluster microtubules, resulting in overstable bundled microtubules (see Fig. 2c for schematics). In this context, it might be worthwhile mentioning that both spastinKO and TauKO mice display late and moderate motor deficits [13, 36, 41, 47, 70], possibly due to stalled microtubule-based transport. In neurons derived from spastinKO-mice or HSP-patients, axonal varicosities that contain organelles and microtubule fragments can be observed [19, 26, 70]. Similar varicosities were also observed in axons of TauKO mice when additional stressors (overexpression of mutAPP or traumatic brain injury) were present, but microtubule content was not investigated. Many different conditions may lead to late and moderate motor deficits, but this raises the question of possible shared mechanisms of spastin and Tau action on maintaining axonal health. In the following section we explore if and how inhibitors of spastin or upstream regulators of spastin could be beneficial for AD and SPAST-caused HSP.

**Table 1:** Effects on microtubules in AD, AD models, models of tauopathy and SPAST-HSP models

Disease model	Effects on microtubules	Suggested cause	Therapeutic/mechanistic intervention	Reference
AD patient study	microtubule reduction	-	-	[10]
AD model: Primary neurons exposed to oligomeric Aβ	specific loss of dendritic microtubules	spastin/TTLL6 mediated aberrant severing caused by Tau missorting and phosphorylation	Taxol, MARK2 overexpression, spastin suppression	[78, 80, 81]
AD model: Primary neurons exposed to prefibrillar Aβ	loss of microtubules	N-terminal domain of Tau (aa 1–248)	-	[37]
Tauopathy model: tg-mice with 0N3R Tau with a pathological mutation (P301L)	decreased microtubule stability	-	Epothilone D	[82]
Model of HSP: tg-mice (putative KO) and derived primary neurons	decreased number of dynamic microtubules; progressive axonopathy	axonal swellings	Nocodazole, Taxol, Vinblastine	[19, 70]
Model of HSP: patient derived olfactory neurons	low levels of acetylated tubulin, slow peroxisome trafficking	-	taxol, vinblastine, epothilone, noscapine	[18]
Model of HSP: Overexpression of mutated spastin	decreased microtubule dynamics	binding of severing deficient spastin isoform M1 to microtubules	reduction of M1 isoform; M85 isoform had no effect	[62, 63]
Model of HSP: iPSC with SPAST mutations	neurite swellings with disrupted microtubules	-	Overexpression of spastin isoforms M1 and M87	[26]
Model of HSP: iPSC cells and knockdown hESCs	increased levels of acetylated tubulin	-	vinblastine	[14]
Model of HSP: tg-mice (putative KO)	-	axonal swellings, perturbed transport	-	[36]
Model of HSP: zebrafish KD of spastin	impaired MT dynamics	spastin function required for physiological MT dynamics	no beneficial effect of nocodazole	[9]
Drosophila model of axon regeneration after axotomy in heterozygous KO and spastin overexpression	no effect on microtubule polarity or developmental neuritic outgrowth	heterozygous KO of spastin impairs axonal regeneration	overexpression of spastin also results in impaired axonal regeneration	[66]

### Similar upstream mechanism of spastin recruitment in AD and HSP: increased polyglutamylation of microtubules

The steps upstream of spastin recruitment in AD and HSP might be similar in spite of the different outcomes: In case of pathological severing as in the proposed model for AD, microtubules first need to be polyglutamylated (by TTLL6 and possibly also by TTLL11 and the priming ligases TTLL1/5) [38]. In case of aberrant microtubule bundling and impaired severing as proposed for HSP, polyglutamylation still might be necessary to guide the severing-deficient spastin to microtubules, but this question has not yet been addressed experimentally. Thus, targeting binding of spastin to microtubules, or microtubule polyglutamylation by TTLL1, 5, 6 or 11 could be a therapeutic option not just for AD and other tauopathies, but also for SPAST caused HSP.

### Pathological gain of function of spastin in HSP: potential benefits of spastin inhibition in HSP

Most mutations of spastin are present within the AAA-ATPase domain and lead to a loss of microtubule severing, therefore HSP caused by spastin mutation is considered a loss of function or haploinsufficiency phenotype [60]. But loss of function of the severing activity of spastin cannot explain important aspects of the disease and observations made in model systems of HSP. There is no correlation between spastin levels and the severity of neurodegeneration in disease and model systems [60, 76]. Knockout or truncation of spastin in mice only results in late and moderate motor deficits, which are a lot more subtle than those in human patients suffering from HSP [36, 70].

Also, HSP linked to spastin mutation affects mainly the corticospinal tracts without any involvement of the peripheral nervous system. In the central nervous system effects are most obvious in the spinal cord. Other parts of the central nervous system are not affected or only at later stages. There are, however, reports of dementia and cognitive decline associated with HSP, but these are very subtle, usually below clinical thresholds, and virtually absent when compared to intrafamilial controls [22, 68], but this is a matter of debate (see e.g. [46]). There is one report on a mutation of spastin (exon 10, R424G), in which authors also looked for other brain pathologies and identified Lewy bodies and tauopathy [73]. The tauopathy found corresponded to Braak stage 2, an AD neuropathology stage which is normally not associated with dementia, but this HSP patient did suffer from dementia [6, 73]. More human studies are needed to test the involvement of Tau in HSP.

Because a loss of function cannot well explain many of the phenotypes of HSP and model systems thereof, a pathological gain of function mechanism which also explains the spinal cord involvement has been proposed [62, 70]. The two major isoforms of spastin resulting from the two start codons show differential distribution and pathology involvement in model systems. M1 is only present in the adult spinal cord, while M87 is ubiquitous; M1 appears to be detrimental to axonal transport and MT dynamics, whereas M87 is not [64]. Also, in humans, significant levels of M1 were only detected in the adult spinal cord, not in the brain [63]. Only mutated M1, but not M87, decorates MTs and thereby could over-stabilize them, resulting in impaired (i.e. reduced) MT dynamics [62]. This mechanism has only been shown for some mutations, but as most of the mutations are present within the AAA-ATPase domain, spastin would retain its ability to bind to and bundle microtubules. Therefore, an inhibitor of M1 spastin activity or M1 binding to MTs could be of therapeutic value for at least some SPAST mutation based cases of HSP.

Spastin has diverse cellular function apart from MT-severing, many of which could result in a pathological gain of function if SPAST is mutated (for review see Baas and Solowska [61]). The M1 isoform specifically interacts with atlastin 1, a large integralmembrane GTPase, which mediates homotypic fusion of ER-tubules. Of note, mutations of atlastin 1 are the second most common cause of HSP [83], indicating that impaired ER-shaping along microtubules is of great importance in HSP. As most SPAST mutations do not affect the interaction motif of spastin for atlastin, impairment of ER-tubule shaping could also be a toxic gain of function mechanism in HSP independent of MT-severing. In a model system of mutated atlastin 1 (encoded by ATL1, also SPG3A), however, impairments could be prevented by the use of microtubule stability modulating drugs (vinblastine and taxol; [84]). This indicates that also in HSP cases that are not caused by mutation of spastin, modulation of microtubule stability could be of therapeutic value, but more studies are needed here to elucidate the molecular pathomechanisms.

### Increased spastin activity in glioblastoma

Of note, increased spastin levels have been linked to increased malignancy and invasiveness of glioblastomas. Although the mechanisms are unclear, increased spastin expression resulted in the formation of protrusions and decreased cell proliferation of cancer cell lines. High-grade malignancy glioblastomas were linked to increased expression of spastin. Spastin could shift cancer populations from cancer cells showing high proliferation to cells that proliferate less, but show greater malignancy and invasiveness. This change in properties of cancer cells results in cancers more difficult to treat. Therefore spastin inhibitors might also be of therapeutic value for certain forms of brain cancer [16].

### Counterindications for the use of spastin inhibitors

Physiological functions of spastin might be essential for cellular homeostasis, which would tamper hope of using spastin as a therapeutic target. Knocking down spastin during early stages of development results in impaired dendritic branching and outgrowth in cell culture [53]. However, there are no reports of developmental defects in human patients of HSP, indeed disease onset usually occurs after several asymptomatic decades of life. Loss of spastin function by knockout in mice only results in late and moderate motor deficits and no developmental deficits [36, 70].

In the context of axon regeneration, it has been reported that axotomized Drosophila sensory neurons are able to regenerate their axon from the axon stump or the dendrite only when spastin is present homozygously, but not when one allele is missing. Other microtubule severing enzymes (e.g. kat-60 (the Drosophila homologue of human katanin-p60), kat-60 L and fidgetin) did not have an effect on axon regeneration in case of heterozygous loss of function mutants. Other microtubule related functions, such as axonal outgrowth during development or microtubule polarity in axons and dendrites were unaltered in the same study [66]. Thus, at least in the context of axon regeneration, lowering spastin activity would not be beneficial.

Taken together, these notions indicate that inhibiting spastin might not harm overall neuronal function and thus could be beneficial for patients. If the gain of function mechanism proves valid for HSP, inhibiting specifically the microtubule severing function of spastin might not be of therapeutic value for HSP patients, as this would lead to even more MT bundling and overstabilization. For the treatment of HSP a microtubule binding inhibitor would be preferable, while for AD either an inhibitor of microtubule severing or microtubule binding inhibitor could be of therapeutic value. It remains unclear, however, whether microtubule bundling by mutated spastin is an in vitro artefact as this has not been demonstrated in vivo. Inhibiting mutant spastin could be beneficial over absence of spastin, but this has not been addressed experimentally.

### Regulation of spastin

Apart from polyglutamylation of microtubules by TTLL6 and TTLL11 (see above), little is known about the regulation of spastin activity in physiological and pathological settings. PAK3 is one of the six known p21-activated kinases (PAKs), which is part of the group I PAKs (PAK1,2,3). PAKs can be activated by multiple signaling molecules, particularly by Rho family small GTPases. In a neuronal context PAKs regulate neurite outgrowth, spine morphology and synaptic plasticity. Mutations in the PAK3 gene were linked to X-linked mental retardation (for review on physiology and pathology of PAKs see [45]). Interestingly, overexpression of PAK3 was found to enhance the Drosophila eye degeneration phenotype induced by overexpression of spastin. Conversely, loss of PAK3 function prevented spastin mediated defects in synaptic function. This indicates a tight genetic and functional interaction between spastin and PAK3 [50]. However, pharmacological manipulation of PAK3 with effects on spastin has not yet been demonstrated.

The Notch signaling agonist Jagged1 was found to induce increased microtubule stability, similar to low doses of the microtubule stabilizer Taxol. Jagged1 downregulates the expression of spastin, which results in higher stability of microtubules [20]. Conversely, exposure of primary neurons to Aβ induced the expression of the Notch antagonist Numb, which could be counteracted by the pro-survival Notch pathway induced by the endocannabinoids anandamide and 2-arachidonoglycerol. As a Notch signaling modulator, anandamide also shifts gamma-secretase processing from APP to Notch1, which would also be beneficial in an AD-setting ([69], see review for Notch signaling and its influence on microtubule stabilization and synaptic plasticity [4]).

Transcription of spastin was shown to be upregulated by the transcription factors NRF1 and SOX11, whereas miR-96 and miR-182 downregulate both mRNA stability and protein levels of spastin [27].

### Modulating microtubule dynamics as therapeutic approaches for AD and HSP

For AD, tauopathy and HSP models, both microtubule stabilizing and destabilizing drugs have been investigated and reported to show beneficial effects (see e.g. [14, 18, 19], and Table 1 for details and complete list). It is surprising that antagonizing drugs can lead to a similar end result, i.e. rescue from SPAST caused defects. The underlying mechanisms are unclear. Microtubule destabilizing agents used (nocodazole, noscapine, vinblastine) might compensate for decreased severing of mutant spastin by increasing the amount of dynamic labile microtubules, and by increasing microtubule mass by minus end detachment of microtubules from their organizing centers [75]. Microtubule stabilizing drugs (taxol, epothilone D) could rely on activation of other microtubule severing enzymes to compensate for decreased severing by mutant spastin. For instance, increased microtubule stabilization leads to increased acetylation of microtubules, which in turn leads to increased katanin based severing [67]. Microtubule-stabilizing drugs such as taxol and epothilone D are used in cancer therapy, as they overstabilize microtubules and thus induce mitotic arrest and apoptosis of dividing cells. At lower concentrations these drugs marginally stabilize microtubules just enough to counteract microtubule destabilization in models of AD, tauopathy and surprisingly also HSP (Table 1 and for review [8]). However, taxol is not blood–brain barrier penetrant and causes peripheral neuropathy, and epothilone D inhibits the P-glycoprotein (Pgp)-transporter (a widely distributed toxin/drug efflux ABC-transporter), both of which could be responsible for undesired neurotoxicity and/or drug-drug interaction. Other promising, non-naturally occurring microtubule-stabilizing drugs are being developed and tested [44].

Using microtubule stabilizing drugs for the treatment of AD and HSP has also some caveats: Common side effects of taxol (when used at elevated concentrations in cancer therapy) include peripheral neuropathy, likely due to impairment of microtubule based transport in the long peripheral neurons (for review see [58]). As dementia patients would have to be treated for decades, even the use of lower concentrations of microtubule stabilizing drugs might result in similar problems.

A clinical trial for epothilone D for the treatment of patients with mild AD was discontinued after a phase 1 trial in 2013. Also, one clinical trial aiming at microtubule stabilization with Davunetide (alias Neuroactive Peptide NAP, an eight amino acid neuropeptide) for the treatment of neurodegeneration (such as the tauopathy Progressive Supranuclear Palsy, PSP) was negative in all endpoints, which prompted the halt of other studies with the same compound for other applications (biomarkers) and dementia (Frontotemporal lobar degeneration (FTLD), Corticobasal Degeneration (CBD); for more information on ongoing and halted clinical trials on AD see www.alzforum.org/clinical-trial-registries). Another microtubule stabilizing drug, TPI287, a synthetic taxane derivative that crosses the blood brain barrier, is used in a clinical phase I safety trial for several tauopathies (AD, CBD, PSP, and 4 Repeat Tauopathy (4RT)) as well as different brain cancer conditions (for more information on clinical trials of TPI287 see www.clinicaltrials.gov).

However, indirect microtubule-stabilization via inhibition of MT-severing by TTLLs and spastin (or other microtubule severing proteins) might be more feasible as these enzymes are present in far lower concentration than tubulin. Very recently, the microtubule severing enzyme fidgetin was shown to trim the growing ends of dynamic microtubules [42]. This implies that after spastin severing, microtubule fragments might be unable to grow due to fidgetin action (see Table 2 for complete list of proteins possibly involved in AD and HSP related microtubule disruption. Considering (i) the past failures of clinical trials on AD with microtubule stabilizing drugs, (ii) the possible gain of function mechanisms in HSP, and (iii) the possible detrimental action of spastin and fidgetin, it becomes clear that we need a better understanding of microtubule dynamics in neurodegeneration. A molecular toolbox of small molecule inhibitors of microtubule severing enzymes would serve the further investigation of pathomechanisms in AD, related tauopathies and HSP, and facilitate the development of new MT-based therapeutics.

**Table 2:** Proteins possibly involved in microtubule breakdown after Aβ insult

Protein (gene name)	Physiological function	Pathological function	Genetic loci [12]
Tau (MAPT)	microtubule stabilization, axonal outgrowth	aberrant transport of proteins (e.g. TTLL6, fyn), f-actin dissassembly	17q21.31
TTLL6 (TTLL6)	polyGlu chain elongation, preferentially at α-tubulin	excessive polyglutamylation leading to aberrant spastin activation	17q21.32
spastin (SPAST)	microtubule dynamics, transport of membrane vesicles/endosomes, dendrite branching	microtubule breakdown	2p22.3
MARK1,2,3,4 (MARK1,2,3,4)	transport regulation via phosphorylation of MAPs	aberrant phosphorylation of MAPs resulting in decreased microtubule protection	1:1q41
			2:11q13.1
			3:14q32.32
			4:19q13.32
TTLL11 (TTLL11)	polyGlu chain elongation, preferentially at α-tubulin	similar action as TTLL6 (but path. role not yet shown)	9q33.2
TTLL1 (TTLL1)	polyGlu chain initiation at α- and β- tubulin	required for action of TTLL6 and TTLL11 (but path. role not yet shown)	22q13.2
CCP1,4,5,6 (AGTPBP1, AGBL1, AGBL5, AGBL4)	remove (poly)glutamate side chains from microtubules	impaired function could result in increased polyglutamylation (but path. role not yet shown)	1: 9q21.33
			4: 15q25.3
			5: 2p23.3
			6: 1p33
Katanin (KATNA1)	microtubule severing, axon branching	also activated by polyglutamylation of microtubules (but path. role not yet shown)	6q24.3
Fidgetin (FIGN)	microtubule severing, mitosis regulation	path. role not shown	2q24.3

## Conclusion

A key result is the identification of a novel reaction cascade implicated in the missorting of the Tau protein in neurons affected by AD. The results reveal a multi-step pathway leading from the exposure of neurons to Aβ oligomers towards Tau-dependent spine loss and synaptic deficits. We identified new contributors and functions in the pathological cascade of AD, centered around missorting of Tau into the somatodendritic compartment. Missorting of Tau causes the loss of dendritic microtubules mediated by TTLL6 and spastin. The reaction cascade provides an explanation for the well-known reduction of microtubules in AD and might contribute to neurodegeneration in other Tau-dependent pathological states. Downregulation of spastin prevents loss of microtubules and missorting of Tau, two key events in Alzheimer pathology. This indicates that spastin or upstream regulators of spastin activity could serve as therapeutic targets for AD and related tauopathies. Moreover, in HSP the pathological gain of function of spastin could be counteracted by targeting the upstream mechanism of spastin recruitment to microtubules, i.e. (i) by inhibiting polyglutamylation by TTLLs or (ii) by increasing the deglutamylation by CCPs, or (iii) by targeting the binding of spastin to microtubules. Future studies will have to show whether microtubule stabilizers, Notch activators, spastin inhibitors, or its upstream regulators have therapeutic value for AD and HSP.

## Abbreviations

AD: Alzheimer disease
AGTPBP1: ATP/GTP Binding protein1-gene name of TTL
AGBL1: ATP/GTP Binding Protein-Like-Gene name of TTLL
CCP: Cytosolic carboxidase(s)
HSP: Hereditary spastic paraplegia
MAP: Microtubule associated protein
MARK: Microtubule affinity regulating kinase
PAK: p21-activated kinase
SPAS: Gene name of spastin, also known as spg4
TTLL: Tubulin tyrosine ligase-like enzyme
